# A randomized clinical study comparing trabectedin combined with regional hyperthermia with trabectedin in patients with advanced soft tissue sarcoma: HyperTET, a German Interdisciplinary Sarcoma Group trial

**DOI:** 10.1016/j.esmoop.2026.106921

**Published:** 2026-04-09

**Authors:** G.-E. Schuebbe, Y. Xu, L.H. Lindner, D. Di Gioia, S. Abdel-Rahman, W.G. Kunz, T. Roshchyna, R. Fenderle, V. Bücklein, P. Reichardt, D. Pink, A. Flörcken, O. Ott, R. Fietkau, E. Kampmann, M. von Bergwelt-Baildon, B. Kasper, R. Issels

**Affiliations:** 1 Department of Medicine III, University Hospital, LMU Munich, Munich, Germany; 2 Institute of Medical Informatics, Biometry, and Epidemiology, LMU Munich, Munich, Germany; 3 Department of Radiology, University Hospital, LMU Munich, Munich, Germany; 4 Helios Klinikum Berlin-Buch, Berlin, Germany; 5 Department of Haematology, Oncology, HELIOS Klinikum Bad Saarow, Bad Saarow, Germany; 6 Klinik für Innere Medizin C der Universitätsmedizin Greifswald, Greifswald, Germany; 7 Department of Hematology, Oncology and Tumorimmunology, Charité Universitätsmedizin Berlin, Berlin, Germany; 8 Department of Radiation Oncology, University Hospital Erlangen, Erlangen, Germany; 9 Comprehensive Cancer Center LMU Munich, Munich, Germany; 10 Mannheim Cancer Center, Mannheim University Medical Center, University of Heidelberg, Mannheim, Germany

**Keywords:** soft tissue sarcoma, randomized trial, trabectedin, regional hyperthermia, regional hyperthermia as tumor-targeting therapy, number of cycle dependency

## Abstract

**Background:**

The German Interdisciplinary Sarcoma Group (GISG) assessed efficacy and safety of trabectedin plus regional hyperthermia (Tr + RHT) versus trabectedin (Tr) in patients with advanced soft tissue sarcoma (STS).

**Patients and methods:**

In this randomized, open-label, multicenter study, adults with advanced STS who had progressed after at least one line of anthracycline-based chemotherapy or were unsuited for this treatment were enrolled. Patients were randomly assigned 1 : 1 to receive Tr + RHT or Tr every 3 weeks. Stratification factors included histological subtype, prior surgery (at any time), metastatic status, and Eastern Cooperative Oncology Group performance status. The primary endpoint was progression-free survival (PFS) analyzed in the intention-to-treat (ITT) population.

**Results:**

Between 19 December 2014 and 7 December 2021, 118 eligible patients from five GISG centers were allocated to Tr + RHT (*n* = 60) or Tr (*n* = 58). Patients received a median of three cycles [interquartile range (IQR) 2-6.2 cycles] in the Tr + RHT arm and four cycles (IQR 2-6 cycles) in the Tr arm. Median PFS was 3.0 months [95% confidence interval (CI) 2.5-5.0 months] with Tr + RHT versus 3.5 months (95% CI 2.8-5.9 months) with Tr (stratified HR 0.86, 95% CI 0.57-1.29, *P* = 0.459). In a Cox proportional hazards model, patients receiving five or more cycles showed a median PFS of 12.8 months (95% CI 9.8-21.0 months) with Tr + RHT versus 7.8 months (95% CI 6.0-12.6 months) with Tr (stratified HR 0.33, 95% CI 0.13-0.86, *P* = 0.023). Most commonly reported grade 3/4 treatment-related adverse events (AEs) were hematologic, hepatic, and infections. Treatment-related deaths were reported in three patients (5.3%) of the Tr arm.

**Conclusions:**

Adding RHT to trabectedin did not improve PFS. Although the study was negative, a post hoc exploratory analysis of the subgroup of patients receiving ≥5 cycles of Tr + RHT showed an improvement in PFS. The combination had a manageable safety profile, serving as the basis for a subsequent study on maintenance therapy with trabectedin or lurbinectedin plus RHT in advanced STS.

## Highlights

• Adding regional hyperthermia to trabectedin did not improve progression-free survival.
• PFS was improved in the subgroup of patients receiving at least five cycles of trabectedin plus regional hyperthermia.
• The combination with regional hyperthermia had a manageable safety profile and supports future maintenance studies.

## Introduction

### Background

For patients with locally advanced or metastatic nonresectable soft tissue sarcoma (STS), systemic treatment with doxorubicin has remained the standard first-line treatment for >50 years.^1^ Until recently, no randomized trial had formally demonstrated a survival advantage of multi-agent chemotherapy over single-agent doxorubicin.^2^ However, the randomized LMS04 trial of the French Sarcoma Group showed that in patients with metastatic or unresectable uterine or soft tissue leiomyosarcoma, induction therapy with doxorubicin plus trabectedin followed by trabectedin maintenance resulted in improved overall survival (OS) and progression-free survival (PFS) compared with doxorubicin alone.^3^

In patients who relapse or progress after first-line therapy, PFS and OS rarely exceeded 6 and 12 months, respectively, before trabectedin became available.^4^ Trabectedin (Yondelis®; PharmaMar, S.A., Madrid, Spain) is approved in the European Union and other countries for the treatment of STS after failure of anthracycline and ifosfamide, or for those patients who are unsuited to receiving these agents.^5^^,^^6^ In addition to its direct cytotoxic effects on malignant cells, trabectedin has selective anti-inflammatory, immunomodulatory, and anti-angiogenic properties.^7^^,^^8^ Heat exposure (40°C-43°C) of cancer cells in preclinical studies and regional hyperthermia (RHT) targeting the tumor area of patients in clinical trials have shown synergistic activity with ionizing radiation or chemotherapy.^9^^,^^10^ Trabectedin’s primary mechanism involves binding to DNA and inducing double-strand breaks (DSBs).^11^ Our prior research showed that heat enhances trabectedin-related cell death in human sarcoma cell lines. The heat-induced molecular mechanisms included a temporary reduced breast cancer 2 (BRCA 2) expression in sarcoma cells, an key protein of the homologous recombination repair (HRR) pathway.^12^ Since HRR-deficient tumors are more susceptible to trabectedin,^13^ we hypothesized that heat-induced HRR deficiency in STS may impair the repair of trabectedin-induced DNA DSBs, thereby enhancing its efficacy.

These findings provided the rationale for the German Interdisciplinary Sarcoma Group (GISG) to initiate the randomized HyperTET trial to assess the efficacy and safety of trabectedin in combination with RHT versus trabectedin alone in STS after disease progression following prior chemotherapy.

## Patients and methods

### Trial design and study oversight

HyperTET was an open-label, prospective, multicenter, randomized trial conducted at five tertiary cancer centers of the GISG, coordinated by the Ludwig-Maximilians-University (LMU), Campus Großhadern, Munich (ClinicalTrials.gov identifier: NCT 02359474). Patients who had failed at least one prior anthracycline-based chemotherapy were randomly assigned in a 1 : 1 ratio to receive either trabectedin combined with RHT (Tr + RHT) or trabectedin alone (Tr), in accordance with the terms of trabectedin’s marketing authorization. Randomization was carried out centrally using a computer-generated allocation system (Randoulette). Patients were stratified based on histotypes into L-STS (liposarcoma or leiomyosarcoma) and non-L-STS (all other sarcoma histological subtypes), previous surgery (yes versus no), presence of metastatic disease (yes versus no) at the time of randomization, and to Eastern Cooperative Oncology Group (ECOG) performance status (0 versus 1/2).

As an open-label trial, treatment assignment was not masked to investigators, patients, or the study sponsor. All study procedures were conducted in accordance with the ethical standards of the 1964 Declaration of Helsinki and its later amendments. The study protocol was approved by the ethics committee of the Ludwig Maximilian University, Munich (8 August 2014), and by the local ethics committee of each participating center. Written informed consent was obtained from all study participants before randomization.

### Patients

Eligible patients were adults (≥18 years) with histologically confirmed soft tissue sarcoma (primary or recurrent), unresectable and/or metastatic relapse or progressive disease (PD), documented by imaging within 4 weeks before treatment. Patients must have received at least one prior line of anthracycline-based chemotherapy or were unsuited to receive this treatment. All patients were required to have measurable disease according to RECIST v1.1,^14^ an ECOG performance status of ≤2, and adequate hematologic, renal, hepatic, and cardiac function.

### Treatments and study procedures

In both treatment arms, trabectedin was administered at the recommended dose of 1.5 mg/m^2^ body surface area via central venous line as a 24-h continuous infusion every 3 weeks (planned dose intensity: 0.5 mg/m^2^/week per cycle). All patients received corticosteroid prophylaxis (e.g. dexamethasone). In the Tr + RHT arm, locoregional hyperthermia (surface or deep, 42°C for a 60-minute period) was applied to a single tumor target area using electromagnetic waves at the end of the 24-h trabectedin infusion. RHT was carried out following the European Society of Hyperthermia Oncology (ESHO) guideline for quality and safety assurance,^15^ using the BSD 2000-3D radiative hyperthermia system (Pyrexar Medical Corporation, Salt Lake City, UT).^16^ A maximum of two dose reductions was permitted if any of the following events occurred during the previous treatment cycle: grade 4 neutropenia lasting for >5 days or associated with fever/infection, increase of bilirubin > upper limit of normal (ULN), alkaline phosphatase >2.5 × ULN, grade 4 thrombocytopenia, alanine aminotransferase/aspartate aminotransferase >2.5 × ULN not reversed to baseline by day 21, or any other grade ≥3 adverse reaction. Upon first occurrence of toxicity, the dose was reduced to 1.2 mg/m^2^ in subsequent cycles. If toxicity recurred, the dose was further reduced to 1.0 mg/m^2^ and maintained in patients with clinical benefit in terms of objective response or disease stabilization. A cycle was considered delayed if administered >6 days after the scheduled date. There was no predefined limit to the number of cycles, treatment continued until PD, unacceptable toxicity, consent withdrawal, or death. Following disease progression, patients were eligible to receive subsequent postprotocol anticancer treatment or supportive care, based on clinical judgment and patient preference.

Radiological response evaluation began at baseline (before the first cycle) and continued until treatment discontinuation for any reason or death. Tumor response evaluations using computed tomography (CT) scans were carried out every 3 months, with the date of disease progression defined by objective PD according to RECIST v1.1.^14^ Imaging was carried out at participating centers and reviewed by an independent radiologist blinded to treatment allocation. All patients were followed for survival until death from any cause or consent withdrawal.

### Endpoints and assessments

The primary endpoint was progression-free survival (PFS), comparing treatment with Tr + RHT to Tr alone. Secondary endpoints included objective response rate (ORR), disease control rate (DCR), overall survival (OS), and safety. PFS was defined as the time from randomization to the earliest occurrence of either disease progression or death (regardless of cause), whereas OS was accounted from the date of randomization until death from any cause. Patients considered lost to follow-up, with no reported disease progression, and alive were censored at the date of last visit. ORR was defined as the proportion of patients who achieved a complete (CR) or partial response (PR). DCR was defined as the proportion of patients with a radiological CR, PR, or stable disease (SD). Adverse events (AEs) were graded according to the National Cancer Institute Common Terminology Criteria for Adverse Events (CTCAE) v4.0 and summarized based on the worst grade 3 or 4 events experienced by ≥20% of patients.

### Statistical analysis

According to existing data for patients receiving trabectedin as second-line chemotherapy we expected a median PFS of 3.3 months in the Tr group. Based on this assumption, to detect [(6-3.3)/3.3]∗100 = 82% increase of PFS in the Tr + RHT group with type I error of 5% and a power of 80%, the final PFS analysis would be carried out when 88 progression or death events had occurred. To compensate a 20% drop-out rate, the total planned sample size was 120 patients. The data cut-off for all analyses was 30 September 2022.

The primary efficacy analysis was conducted on the intention-to-treat (ITT) analysis set, which included all eligible randomly assigned patients, regardless of whether they received the allocated treatment. Safety analyses were based on the safety population, defined as patients who received at least one dose of treatment. Tumor response evaluation included all patients who underwent at least one imaging assessment. The primary endpoint (PFS) was analyzed after the required number of progression and/or death events had occurred, and concerned all other endpoint criteria, including OS evaluation. Descriptive statistics were used to summarize demographic and baseline characteristics of patients. Categorical variables were presented as counts and frequencies, while continuous variables were reported as median [range or interquartile range (IQR)]; significance testing was carried out using Mann–Whitney U test, chi-square test (or Fisher’s exact test), as appropriate. For time-to-event outcomes, survival rate was first estimated using the Kaplan–Meier method and was compared using the stratified log-rank test. Covariates were selected *a priori* based on known prognostic relevance in metastatic soft tissue sarcoma (histological subtype, prior surgery, presence of metastases, ECOG performance status). Treatment effect was estimated using the Cox proportional hazards model, stratifying by histological subtype of sarcoma, prior surgery, metastasis status, and ECOG. For ordinal categorical outcomes, the proportional odds model was used, conditioning on the stratification factors. All tests were two-sided, and significance was accepted at the 5% level. All statistical analyses were done with SAS software and R software.

### Role of the funding source

HyperTET was an investigator-initiated trial supported by PharmaMar. PharmaMar did not participate in the design, collection, analysis, interpretation of data, or any other aspect of the trial. All authors had the final responsibility to submit the manuscript for publication.

## Results

### Patient characteristics

**Figure 1:**
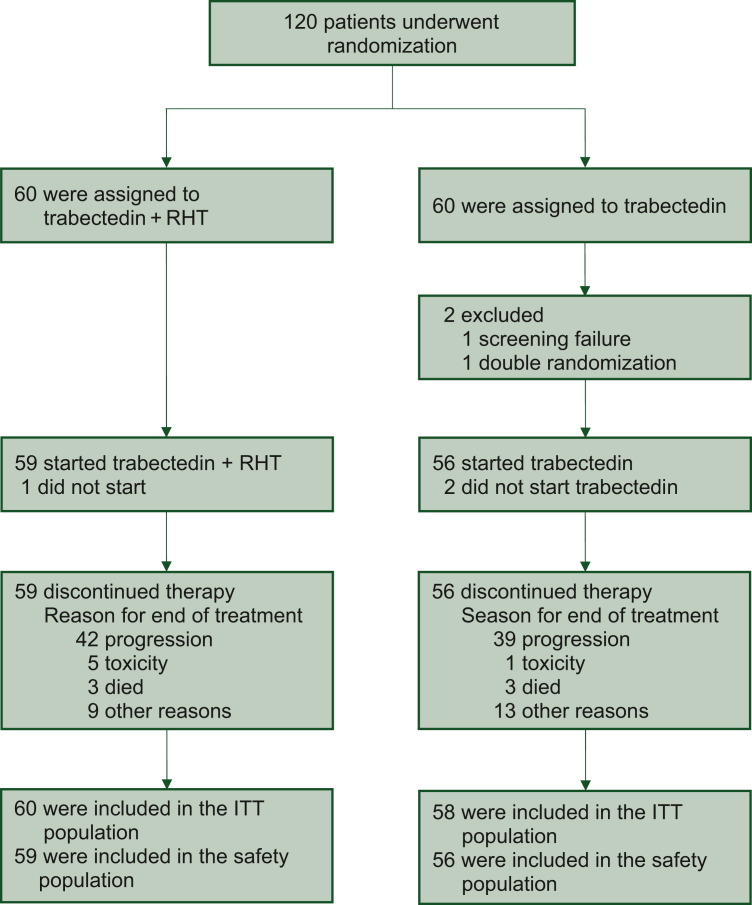
**Randomization and treatment of patients.** ITT, intention-to-treat; RHT, regional hyperthermia.

Between 19 December 2014 and 7 December 2021, a total of 120 patients were enrolled across five GISG centers and allocated to receive Tr + RHT (*n* = 60) or Tr alone (*n* = 60). In the Tr arm, two patients were excluded, one patient due to screening failure and another due to duplicate randomization, resulting in 118 patients included in the efficacy analysis. Because three patients did not start treatment (one in the Tr + RHT, two in the Tr arm), 115 patients were evaluated for safety (Figure 1). Disease progression was the most common reason for treatment discontinuation regardless of treatment group (Tr + RHT 71.2% versus Tr 69.6%). Discontinuation due to toxicity or death without progression occurred in 13.5% of patients in the Tr + RHT group and in 7.1% of the Tr group. Other reasons unrelated to progression in the Tr + RHT versus Tr group included withdrawal of consent (0% versus 7.1%), patient’s wish for treatment interval (7.1% versus 8.5%), investigator decision (5.1% versus 1.8%), lost to follow-up (0% versus 1.8%), and other events (1.7% versus 5.3%), respectively.

Baseline demographics (sex, age, ECOG status) and disease characteristics were well balanced between the Tr + RHT and Tr arms, particularly regarding the number of patients with L-STS (61.7% versus 69.0%) and non-L-STS (38.3% versus 31.0%), and the proportion of patients with metastatic disease (81.7% versus 79.3%) at study entry. Patients were heavily pretreated. In both treatment arms, chemotherapy had been previously administered in the neoadjuvant/adjuvant setting (65.0% versus 55.2%) and combined with hyperthermia (55.0% versus 44.8%), respectively, with 38.3% of patients in the Tr + RHT arm receiving trabectedin as second- to fourth-line treatment versus 41.4% of patients in the Tr arm (Table 1).

**Table 1:** Patient and disease characteristics at baseline

Patients	Trabectedin + RHT *n* = 60	Trabectedin *n* = 58	Total *n* = 118
Sex, *n* (%)			
Male	29 (48.3)	20 (34.5)	49 (41.5)
Female	31 (51.7)	38 (65.5)	69 (58.5)
Age at randomization (years)			
Median (range)	63 (53-71.2)	59.5 (54-70.8)	61 (53.2-71)
Eastern Cooperative Oncology Group performance status, *n* (%)			
0	37 (61.7)	35 (60.3)	72 (61)
1	19 (31.7)	20 (34.5)	39 (33.1)
2	2 (3.3)	1 (1.7)	3 (2.5)
Missing	2 (3.3)	2 (3.4)	4 (3.4)
Sarcoma histology, *n* (%)			
L-sarcoma	37 (61.7)	40 (69.0)	77 (65.3)
Liposarcoma^a^	19 (31.7)	19 (32.8)	38 (32.2)
Leiomyosarcoma	18 (30.0)	21 (36.2)	39 (33.1)
Non-L-sarcoma	23 (38.3)	18 (31.0)	41 (34.7)
Synovial sarcoma	3 (5.0)	3 (5.2)	6 (5.1)
Undifferentiated sarcoma	5 (8.3)	6 (10.3)	11 (9.3)
Angiosarcoma	1 (1.7)	2 (3.4)	3 (2.5)
Other histologies	14 (23.3)	7 (21.1)	21 (17.8)
Histoprognostic grade, *n* (%)			
Grade 1	8 (13.3)	4 (6.9)	12 (10.2)
Grade 2	21 (35.0)	23 (39.7)	44 (37.3)
Grade 3	29 (48.3)	30 (51.7)	59 (50.0)
Missing	2 (3.3)	1 (1.7)	3 (2.5)
Tumor status, *n* (%)			
Metastatic disease	49 (81.7)	46 (79.3)	95 (80.5)
Nonmetastatic disease	11 (18.3)	12 (20.7)	23 (19.5)
Time between first diagnosis and randomization (month)			
Median (range)	23.4 (11.9-48.2)	38 (16.2-89.0)	30 (13.1-66.8)
Prior chemotherapy			
Neoadjuvant/adjuvant, *n* (%)	39 (65.0)	32 (55.2)	71 (60.2)
Advanced, median lines (range)	1 (1-2)	1 (1-2)	1 (1-2)
Type of prior chemotherapy, *n* (%)^b^			
Antracyclines	10 (16.7)	11 (19.0)	21 (17.8)
Ifosfamide ± antracyclines	36 (60.0)	33 (56.9)	69 (58.5)
Dacarbazine (DTIC) ± antracyclines	20 (33.3)	19 (32.8)	39 (33.1)
Gemcitabine + docetaxel	7 (11.7)	8 (13.8)	15 (12.7)
Pazopanib	1 (1.7)	5 (8.6)	6 (5.1)
Others	13 (21.7)	10 (17.2)	23 (19.5)
Previous hyperthermia, *n* (%)			
Hyperthermia	33 (55.0)	26 (44.8)	59 (50.0)
No hyperthermia	27 (45.0)	32 (55.2)	59 (50.0)
Prior surgery, *n* (%)			
Surgery^c^	53 (88.3)	55 (94.8)	108 (91.5)
No surgery	7 (11.7)	3 (5.2)	10 (8.5)

a Includes four patients (6.7%, trabectedin + RHT) and two patients (3.4%, trabectedin) with myxoid liposarcoma.

b Multiple counts.

c Includes one patient (trabectedin + RHT) and two patients (trabectedin) with local tumor who underwent surgery within 4 weeks before randomization.

### Extent of exposure

During the randomized phase of the study, patients in the Tr + RHT arm received a median of 3 cycles (IQR 2-6.2 cycles), and patients in the Tr arm received a median of 4 cycles (IQR 2-6 cycles). Similar proportions were noted for patients in both treatment arms receiving >6-9 cycles (8.3% versus 8.6%) and >9-12 cycles (5.0% versus 8.6%), except for >12 cycles, which was almost twice as high in the Tr + RHT arm (11.7% versus 6.9%). Cycle delays or dose reductions occurred in 12.0% and 13.6% of patients in the Tr + RHT group compared with 7.9% and 22% of patients in the Tr group, respectively. In the Tr + RHT group, a total of 316 cycles were administered over a median treatment duration of 12.0 weeks (range 6.9-23.5 weeks). In the Tr group, a total of 305 trabectedin cycles were administered over a median treatment duration of 11.9 weeks (range 7.4-26.8 weeks). Most patients in both arms received ≥80% of the planned dose (Tr + RHT 84.8% versus Tr 89.5%) (Table 2). Poststudy chemotherapies were comparable between both treatment arms and included gemcitabine plus docetaxel, pazopanib, ifosfamide, eribulin and rechallenge with anthracycline or trabectedin with/without RHT beyond progression, administered at the investigator’s discretion.

**Table 2:** Treatment exposure

Treatment delivery	Trabectedin + RHT *n* = 60	Trabectedin *n* = 58	Total *n* = 118
Time on treatment (weeks)			
Median (range)	12 (6.9-23.5)	11.9 (7.4-26.8)	11.9 (6.9-24.7)
Cycles per patient, *n* (%)			
Median (interquartile range)	3 (2-6.2)	4 (2-6)	3.5 (2-6)
0	1 (1.7)	2 (3.4)	3 (2.5)
1	4 (6.7)	7 (12.1)	11 (9.3)
2	17 (28.3)	9 (15.5)	26 (22)
3	9 (15)	10 (17.2)	19 (16.1)
4	11 (18.3)	9 (15.5)	20 (16.9)
5	0 (0)	4 (6.9)	4 (3.4)
6	3 (5)	3 (5.2)	6 (5.1)
>6	5 (8.3)	5 (8.6)	10 (8.5)
>9	3 (5)	5 (8.6)	8 (6.8)
>12	7 (11.7)	4 (6.9)	11 (9.3)
Relative dose intensity, *n* (%)			
<80%	48 (15.2)	32 (10.5)	80 (12.9)
≥80%	268 (84.8)	273 (89.5)	541 (87.1)
Total cycles, *N*	316	305	621
Dose modification and cycles delayed (per cycle), *n* (%)			
Cycles susceptible to have dose modification or delay	316	305	621
No dose modification or cycle delay	187 (59.2)	179 (58.7)	366 (58.9)
Dose modification and cycle delay	48 (15.2)	35 (11.5)	83 (13.4)
Cycle delay only	38 (12)	24 (7.9)	62 (10.0)
Dose modification only	43 (13.6)	67 (22.0)	110 (17.7)
Main reasons for cycle delay per cycle; susceptible cycles	86	59	145
Hematologic toxicity	21 (24.4)	8 (13.6)	29 (20.0)
Hepatic toxicity	5 (5.8)	3 (5.1)	8 (5.5)
Patient wish	14 (16.3)	20 (33.9)	34 (23.4)
Infection	15 (17.4)	4 (6.8)	19 (13.1)
Other reasons	27 (31.4)	16 (27.1)	43 (29.7)
Unknown	4 (4.7)	8 (13.6)	12 (8.3)
Main reasons for dose reduction per cycle; susceptible cycles	91	102	193
Hematologic toxicity	15 (16.5)	6 (5.9)	21 (10.9)
Hepatic toxicity	15 (16.5)	31 (30.4)	46 (23.8)
Patient wish	0 (0)	2 (2.0)	2 (1.0)
Renal insufficiency	3 (3.3)	0 (0)	3 (1.6)
Neuropathy	8 (8.8)	4 (3.9)	12 (6.2)
Infection	4 (4.4)	0 (0)	4 (2.1)
Other reasons	40 (44.0)	50 (49.0)	90 (46.6)
Unknown	6 (6.6)	9 (8.8)	15 (7.8)
End of treatment, *n* (%)			
Yes	60 (100)	58 (100)	118 (100)
Further chemotherapy, *n* (%)^a^			
Gemcitabine + docetaxel	15 (50)	17 (56.7)	32 (53.3)
Pazopanib	11 (36.7)	7 (23.3)	18 (30)
Ifosfamid	2 (6.7)	1 (3.3)	3 (5)
Eribulin	4 (13.3)	5 (16.7)	9 (15)
Other	12 (40)	15 (50)	27 (45)
Anthracycline rechallenge	4 (66.7)	6 (54.5)	10 (58.8)
Trabectedin rechallenge/beyond progression	2 (33.3)	5 (45.5)	7 (41.2)
Further radiotherapy, *n* (%)			
Radiotherapy	16 (26.7)	14 (24.1)	30 (25.4)
No radiotherapy	44 (73.3)	44 (75.9)	88 (74.6)
Further surgery, *n* (%)			
Surgery	16 (26.7)	16 (27.6)	32 (27.1)
No surgery	44 (73.3)	42 (72.4)	86 (72.9)

a Multiple counts.

### Efficacy

**Figure 2:**
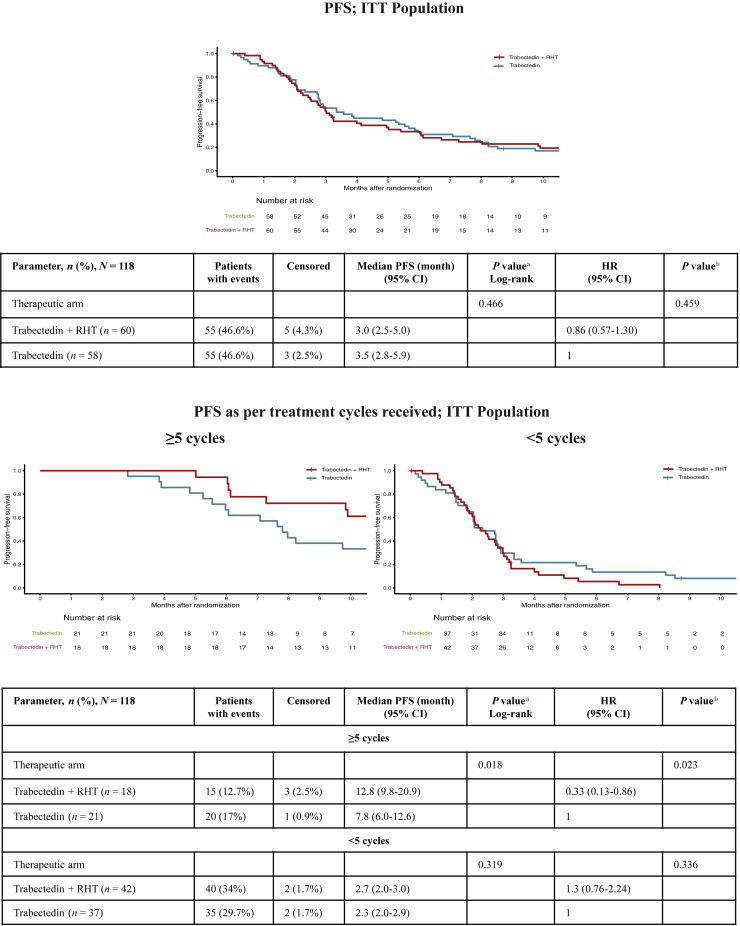
**Kaplan–Meier plots of progression-free survival (PFS) by independent radiology review.** CI, confidence interval; HR, hazard ratio; ITT, intention-to-treat; NA: not assessable; RHT, regional hyperthermia. ^a^Log-rank by treatment. ^b^The hazard ratio for trabectedin versus trabectedin + RHT was estimated using a Cox proportional hazard model, stratified by the histological subtype [liposarcoma or leiomyosarcoma soft tissue sarcoma (L-STS) versus non-L-STS], surgery (yes versus no), metastasis (yes versus no), and Eastern Cooperative Oncology Group status (0 versus 1 or 2).

At the time of the primary endpoint analysis, 110 PDs or death events (93.2%) were recorded, whereas 8 patients (6.8%) who were alive without confirmed PD were censored. After median follow-up of 21.4 months, median PFS was 3.0 months [95% confidence interval (CI) 2.5-5.0 months] in the Tr + RHT arm versus 3.5 months (95% CI 2.8-5.9 months) in the Tr arm [stratified hazard ratio (HR) 0.86, 95% CI 0.57-1.30, *P* = 0.459] (Figure 2; upper part). Univariate analyses demonstrated consistent PFS outcomes across subgroups with no statistically significant differences observed. A notable trend favoring the Tr + RHT arm was seen in patients (*n* = 39) who received five or more cycles, although this did not reach statistical significance (HR 0.60, 95% CI 0.30-1.18) (Supplementary Figure S1, available at https://doi.org/10.1016/j.esmoop.2026.106921). In the multivariate Cox proportional hazards model stratified by the histological subtype of sarcoma, prior surgery, metastasis, and ECOG status, the benefit for this subgroup was significant. Among patients who received five or more cycles, median PFS was 12.8 months (95% CI 9.8-20.9 months) in the Tr + RHT arm versus 7.8 months (95% CI 6.0-12.6 months) in the Tr arm (stratified HR 0.33, 95% CI 0.13-0.86, *P* = 0.023). For patients who received fewer than five cycles, median PFS was 2.3 months (95% CI 2.0-3.0 months) in the Tr + RHT arm versus 2.3 months (95% CI 2.0-2.9 months) in the Tr arm (stratified HR 1.30, 95% CI 0.76-2.24, *P* = 0.336) (Figure 2; lower part). To test the independence of prior surgery with the presence of metastatic disease, we conducted the Fisher’s exact test for the ITT population (*n* = 118). No statistically significant association was observed between surgery and the presence of metastases in the ITT population (Fisher’s exact test two-sided *P* = 0.21) and in the subgroup (*n* = 39) with five or more cycles (Fisher’s exact test two-sided *P* = 0.56). Baseline characteristics and treatment exposure were well balanced between both arms among patients who received five or more cycles. When comparing the parameters age, histological subgroups, grade, tumor status, and type of previous treatment, there was no difference. Patients received a median of 11 cycles (range 8-15 cycles) in the Tr + RHT arm versus a median of 8 cycles (range 6-12 cycles) in the Tr arm, with twice as many patients in the Tr + RHT arm receiving >12 cycles (Supplementary Table S1, available at https://doi.org/10.1016/j.esmoop.2026.106921).

**Figure 3:**
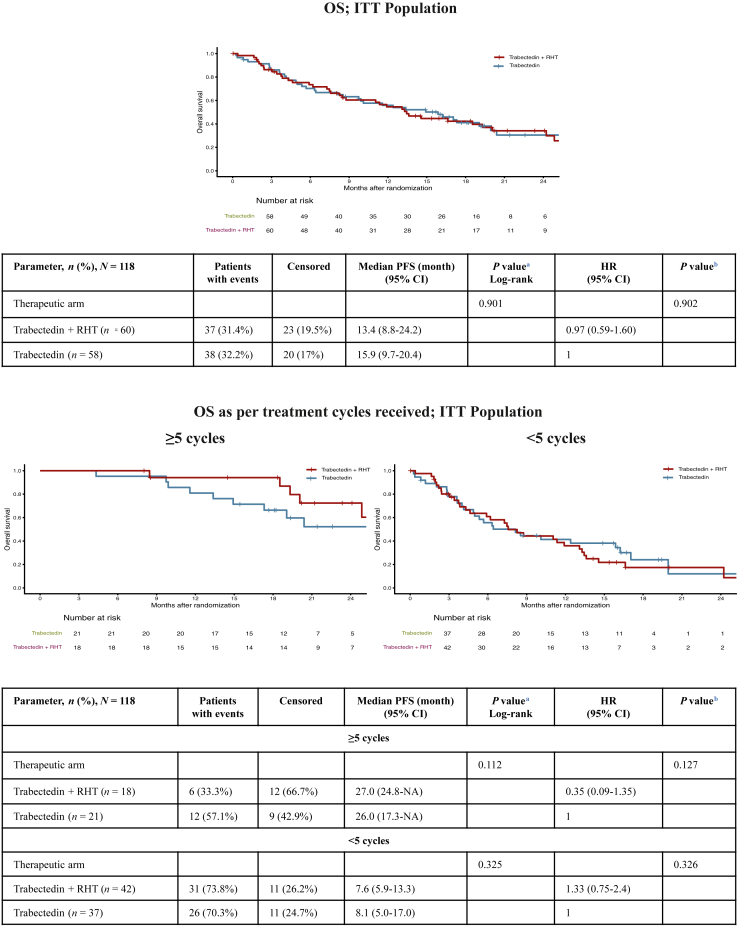
**Kaplan–Meier plots of overall survival.** CI, confidence interval; HR; hazard ratio; ITT, intention-to-treat population; RHT, regional hyperthermia. ^a^Log-rank by treatment. ^b^The hazard ratio for trabectedin versus trabectedin + RHT was estimated using a Cox proportional hazard model, stratified by the histological subtype [liposarcoma or leiomyosarcoma soft tissue sarcoma (L-STS) versus non-L-STS], surgery (yes versus no), metastasis (yes versus no), and Eastern Cooperative Oncology Group status (0 versus 1 or 2).

After 75 death events, median OS was 13.4 months (95% CI 8.8-24.2 months) for the Tr + RHT arm versus 15.9 months (95% CI 9.7-20.4 months) for the Tr arm (stratified HR 0.97, 95% CI 0.59-1.61, *P* = 0.902) (Figure 3; upper part). For patients who had received five or more cycles, median OS was 27.0 months [95% CI 24.9 months-not assessable (NA)] in the Tr + RHT arm versus 26.0 months (95% CI 17.3 months-NA) in the Tr arm (stratified HR 0.35, 95% CI 0.09-1.35, *P* = 0.127). In patients with fewer than five cycles, median OS was 7.6 months (95% CI 5.9-13.3 months) in the Tr + RHT arm versus 8.1 months (95% CI 5.0-17.1 months) in the Tr arm (stratified HR 1.33, 95% CI 0.75- 2.37, *P* = 0.326) (Figure 3; lower part).

No CRs were observed in either treatment group. While two patients achieved a PR reaching a disease control rate (DCR) of 46.9% among 49 assessable patients of the Tr + RHT arm, two patients with PR were observed in 46 assessable patients of the Tr arm reaching a DCR of 56.5%. For patients who received five or more cycles, the DCR was 100% (15/15) in the Tr + RHT arm compared with 95% (19/20) in the Tr arm (Supplementary Table S2, available at https://doi.org/10.1016/j.esmoop.2026.106921).

### Safety

During the randomized phase of the study, the most commonly reported treatment-related grade 3/4 AEs were primarily laboratory abnormalities indicating transient liver toxicity and hematologic toxicity due to myelosuppression (Table 3). Compared with the Tr arm, grade 3/4 liver toxicity was higher in the Tr + RHT arm, with increased transaminases (15.3% versus 10.7%) and elevated γ-glutamyltransferase (18.7% versus 12.5%), whereas grade 3/4 hematologic toxicity was higher in the Tr arm, including anemia (12.5% versus 6.8%), neutropenia (19.6% versus 6.8%) with episodes of febrile neutropenia (7.2% versus 0%), and leukopenia (12.5% versus 6.8%). Apart from laboratory abnormalities, infections were more frequently reported in the Tr + RHT arm (16.9% versus 5.4%), although all were grade 3 events. The incidence of patients who died within 30 days after the last treatment cycle due to tumor progression was similar in both treatment arms (Tr + RHT: six patients; 10.2% versus Tr: five patients; 8.9%) One patient in the Tr arm died from acute respiratory insufficiency. Treatment-related deaths were only reported in the Tr group (3 patients; 5.3%) caused by septic shock (*n* = 2), and chronic renal failure (*n* = 1).

**Table 3:** Most common adverse events, occurring in ≥20% (safety population), *n* (%); adverse events by treatment and grade.

Adverse event	Trabectedin + RHT, *n* (%) *n* = 59				Trabectedin, *n* (%) *n* = 56			
	All grades	Grades 1/2	Grade 3	Grade 4	All grades	Grades 1/2	Grade 3	Grade 4
Liver toxicity	40 (67.8)	19 (32.2)	19 (32.2)	2 (3.4)	33 (58.9)	20 (35.7)	12 (21.4)	1 (1.8)
Increased transaminases	17 (28.8)	8 (13.6)	9 (15.3)	0	15 (26.8)	9 (16.1)	5 (8.9)	1 (1.8)
Elevated γ-glutamyltransferase	16 (27.1)	5 (8.5)	9 (15.3)	2 (3.4)	10 (17.9)	3 (5.4)	7 (12.5)	0
Other (liver toxicity)	7 (11.9)	6 (10.2)	1 (1.7)	0	8 (14.3)	8 (14.3)	0	0
Nausea/vomiting	20 (33.9)	20 (33.9)	0	0	27 (48.2)	26 (46.4)	1 (1.8)	0
Infection (*n* = 1, na)	26 (44.1)	16 (27.1)	10 (16.9)	0	20 (35.7)	16 (28.6)	3 (5.4)	0
Fatigue (*n* = 1, na)	19 (32.2)	16 (27.1)	2 (3.4)	0	19 (33.9)	19 (33.9)	0	0
Anemia	12 (20.3)	8 (13.6)	4 (6.8)	0	18 (32.1)	11 (19.4)	7 (12.5)	0
Neutropenia (*n* = 1, na)	16 (27.1)	11 (18.6)	4 (6.8)	0	14 (25.0)	3 (5.4)	7 (12.5)	4 (7.1)
Febrile neutropenia	0	0	0	0	4 (7.1)	0	2 (3.6)	2 (3.6)
Leukopenia	8 (13.6)	4 (6.8)	4 (6.8)	0	12 (21.4)	5 (8.9)	5 (8.9)	2 (3.6)

na, no data for grade available.

## Discussion

HyperTET represents the first randomized, prospective trial to evaluate whether hyperthermia added to trabectedin compared with trabectedin alone reduces the risk of progression or death in patients with STS after anthracycline-based chemotherapy. Patients studied were heavily pretreated, having experienced failure of previous systemic therapy in the neoadjuvant/adjuvant setting including RHT, prior surgery at any time, and radiotherapy, and they had rapidly progressing disease with a dismal prognosis. The study failed to meet its primary endpoint. In our control arm, trabectedin resulted in a PFS of 3.5 months (95% CI 2.8-5.9 months), which is consistent with a PFS of 3.1 months (95% CI 1.8-5.9 months) observed in the trabectedin arm of the randomized phase III T-SAR trial of the French Sarcoma Group comparing trabectedin with best supportive care, with prolongation of PFS for trabectedin as the primary endpoint (HR 0.39, *P* < 0.001).^17^ The result also applies to the randomized phase II study establishing the efficacy and safety of the 24-h infusion of trabectedin as used in our study with a PFS of 3.6 months.^18^ Assuming the same standard of care in the administration of trabectedin at the GISG centers for a comparable risk group of patients, the outcome if combined with RHT (PFS 3.0 months, 95% CI 2.5-5.0 months) was worse than the assumption to increase PFS to 6.0 months of patients who received additional hyperthermia. The patients of the control arm received a median of four cycles with trabectedin alone versus a median of three cycles with Tr plus RHT in the experimental arm. Consequently, the evaluation of the primary endpoint—superiority of the combination with RHT—was limited to the effect of hyperthermia by only three RHT applications. An important finding of the current study is that in an exploratory analysis of patients who received five or more cycles or fewer than five cycles of therapy, PFS was significantly improved by the addition of five or more RHT applications to trabectedin (median PFS 12.8 months) with a 67% risk reduction for progression or death compared with five or more cycles of trabectedin alone (median PFS 7.8 months) (HR 0.33, 95% CI 0.13-0.86, *P* = 0.023). Such a dose–effect principle in relation to the required therapy cycles combined with RHT corresponds to our results from the EORTC 62961 study in locally advanced STS. When comparing first-line, neoadjuvant chemotherapy with or without RHT, the full course of four preoperative chemotherapy cycles (two RHTs per cycle) was associated with a significant benefit in local PFS and long-term survival for patients who received eight RHT treatments according to the protocol.^19^^,^^20^ This is in line with observations in the meta-analysis of neoadjuvant chemoradiotherapy in combination with RHT.^21^ A clear dependency for clinical success (pathological complete response) on the number of hyperthermia cycles was observed. It was found that the combination only provided a significant benefit when more than four cycles were administered (23% versus 5% pathological complete response rate, *P* < 0.05).^22^

In addition, the complete administration of the combination of neoadjuvant chemotherapy plus RHT, in contrast to chemotherapy alone, induces a positive immunomodulation of the tumor microenvironment, which may also be significant for the effect of trabectedin in combination with RHT because trabectedin behaves as a similar immunomodulatory drug in the subversion of the protumor microenvironment.^23^^,^^24^

Within this context it is important to note that the HyperTET study primarily included patients with metastatic sarcoma, and in these cases, RHT reached only a portion of the tumor sites. We therefore assume that hyperthermia has an effect that goes beyond local tumor control.^25^

In our present study, the predictive meaning of the number of cycles with the hazard for PFS also became evident for the overall study population. When we explored the relationship between the number of cycles and the hazard for PFS, the data allowed the hypothesis that for at least eight cycles, the Tr + RHT group of patients has the potential for an improvement in PFS compared with the Tr group (Supplementary Figure S2, available at https://doi.org/10.1016/j.esmoop.2026.106921).

For trabectedin alone, post hoc analyses of pivotal clinical trials revealed that the number of cycles also plays a significant role in improving PFS. If patients had been treated for more than six cycles, they were able to achieve a significant benefit in long-term tumor control.^26^^,^^27^ Within the compassionate use program (ATU) in France, using the standard 3-weekly regimen of trabectedin, 31% of patients were without progression after an initial six cycles. Patients treated with seven or more cycles had a significantly better PFS (median 5.3 months versus 10.5 months, *P* = 0.001).^26^ In the T-DIS randomized phase II trial, prospectively comparing interruption versus continuation in patients who had not progressed after six cycles, continued therapy with trabectedin was associated with a significant improvement in PFS compared with treatment interruption. Median PFS was 7.2 months in the continuation group compared with 4.0 months in the interruption group (*P* = 0.020).^27^ Our study showed a comparable proportion of patients who were stable with no progression after five or more cycles of trabectedin, observed in approximately one-third of patients in both treatment arms (Tr + RHT 30.5% versus Tr 37.5%). In the current study, no difference was observed in terms of OS between the two arms, nor in an analysis by cycles received. Indeed, our study was underpowered to assess OS, and the study protocol allowed crossover from the Tr to the Tr + RHT arm at progression.

Regarding toxicity, the present study showed an acceptable and manageable safety profile including those patients who remained on therapy for prolonged periods of time. The most frequently reported grade 3-4 AEs were generally transient and noncumulative, and were managed by dose delays, reductions, supportive care, and, if required, treatment discontinuations.

Our study had some limitations. In the multivariate analysis, in addition to well-known prognostic parameters, surgical intervention at any time before randomization (resection after neoadjuvant therapy, metastasectomy) was considered as an additional prognostic parameter. Although no dependence was found between the presence of metastases and prior surgery in our study, the prognostic significance of surgical intervention as an independent parameter of STS patients with metastatic disease was demonstrated in the METASYN and METASARC studies.^28^^,^^29^ Retrospectively, the analysis highlights a key limitation in the study design—namely, no administration of two RHT treatments added to trabectedin per cycle, which hindered a full evaluation of RHT’s role. While the overall cohort showed no benefit from Tr + RHT to the primary endpoint, patients receiving five or more treatment cycles demonstrated improved PFS, suggesting potential benefit with prolonged therapy. Given the limited sample size and *post hoc* nature of this analysis, these findings are exploratory and do not constitute formal evidence of efficacy. However, the observation of sustained disease control without significant toxicity in patients receiving five or more cycles supports further investigation of Tr + RHT as a maintenance approach. Future studies should prospectively assess the continuation of Tr + RHT versus Tr monotherapy beyond six cycles, aligned with the T-DIS trial rationale. Prior evidence from the LM04 study underscores the value of maintenance therapy with Tr and may extend to the better-tolerated agent lurbinectedin.^30^
