# Supplementary material for: A randomized clinical study comparing trabectedin combined with regional hyperthermia with trabectedin in patients with advanced soft tissue sarcoma: HyperTET, a German Interdisciplinary Sarcoma Group trial
Source: ESMO Open. 2026 Apr 9;11(4):106921. doi: 10.1016/j.esmoop.2026.106921 (PMC13091197; doi:10.1016/j.esmoop.2026.106921)
Supplement: Supplementary Figures and Tables [file mmc1.pdf]

SUPPLEMENTARY DATA

Subgroup	Trabectedin	Trabectedin+RHT	HR (95% CI)
All patients	58	60	1.03 (0.71 - 1.50)
Histology			
L-Sarcoma	40	37	1.07 (0.67 - 1.72)
Non-L-Sarcoma	18	23	0.77 (0.40 - 1.48)
Metastasis			
Metastasis	46	49	0.92 (0.60 - 1.40)
No Metastasis	12	11	1.42 (0.59 - 3.41)
ECOG			
Grade 0	37	39	1.06 (0.65 - 1.71)
Grade 1+2	21	21	0.91 (0.49 - 1.70)
Gender			
Male	20	29	0.98 (0.54 - 1.78)
Female	38	31	1.16 (0.70 - 1.91)
Lines of prior chemotherapy			
>2	5	3	2.78 (0.39 - 20.07)
≤2	53	57	1.01 (0.69 - 1.49)
Cycles of chemotherapy during the study			
<5	37	42	1.32 (0.82 - 2.12)
≥5	21	18	0.60 (0.30 - 1.18)

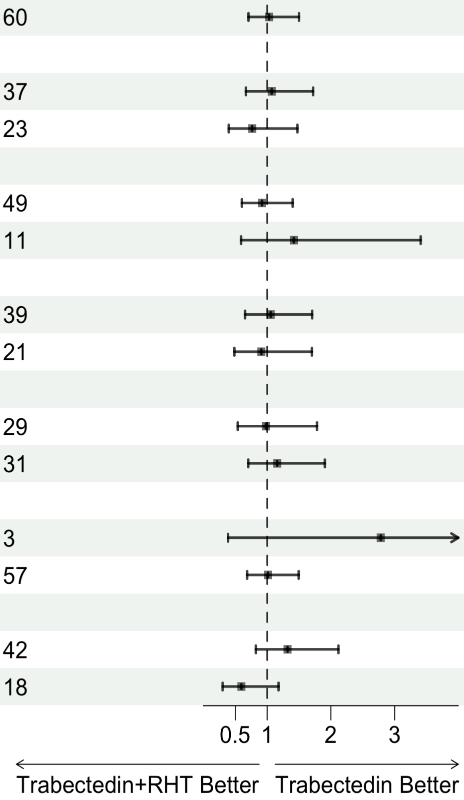

Supplementary Figure 1. Forest plot progression-free survival (ITT population)
Four patients have missing ECOG values and were included in the subgroup with most participants (Grade 0).

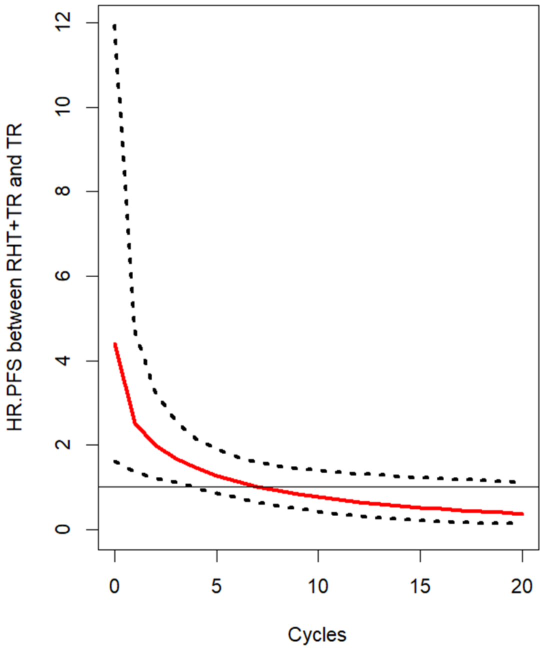

Supplementary Figure 2. Dependency between the hazard ratio (HR Tr+RHT/Tr) of progression-free survival and the number of cycles received (ITT population).
The HR is estimated <1 for 8 cycles or more received (PFS better in the Trabectedin+RHT arm) and >1 for 7 cycles or less received (PFS better in the Trabectedin arm) **(solid red line)**. The black broken lines represent the lower and upper 95% confidence intervals for the estimated functional relationship.

Patients	Trabectedin+RHT n = 18	Trabectedin n = 21	Total *N* = 39
Sex, n (%)			
Male	11 (61.1)	5 (23.8)	16 (41)
Female	7 (38.9)	16 (76.2)	23 (59)
Age at randomization (years)			
Median (range)	62 (53.2-66.5)	61 (51-64)	61 (52.5-66)
Eastern Cooperative Oncology Group performance status, n (%)			
0	13 (72.2)	14 (66.7)	27 (69.2)
1	5 (27.8)	5 (23.8)	10 (25.6)
2	0 (0)	1 (4.8)	1 (2.6)
Missing	0 (0)	1 (4.8)	1 (2.6)
Sarcoma histology, n (%)			
L-sarcoma [a]	13 (72.2)	16 (76.2)	29 (74.4)
Non-L-sarcoma	5 (27.8)	5 (23.8)	10 (25.6)
Histoprognostic grade, n (%)			
Grade 1	2 (11.1)	2 (9.5)	4 (10.3)
Grade 2	9 (50)	9 (42.9)	18 (46.2)
Grade 3	6 (33.3)	10 (47.6)	16 (41)
Missing	1 (5.6)	0 (0)	1 (2.6)
Tumor status, n (%)			
Metastatic disease	15 (83.3)	15 (71.4)	30 (76.9)
Non metastatic disease	3 (16.7)	6 (28.6)	9 (23.1)
Time between first diagnosis and randomization (month)			
Median (range)	53.2 (21.3-71.3)	43.6 (19.3-98.1)	46 (19.2-88.5)
Prior chemotherapy			
Neoadjuvant/adjuvant, n (%)	11 (61.1)	13 (61.9)	24 (61.5)
Advanced, median lines (range)	1 (1-2)	1 (1-2)	1 (1-2)
Type of prior chemotherapy, n (%) [b]			
Antracyclines	6 (33.3)	3 (14.3)	9 (23.1)
Ifosfamide ± antracyclines	11 (61.1)	9 (42.9)	20 (51.3)
DTIC ± antracyclines	2 (11.1)	10 (47.6)	12 (30.8)
Others	6 (33.4)	8 (38.0)	14 (35.9)
Previous hyperthermia, n (%)			
Hyperthermia	10 (55.6)	8 (38.1)	18 (46.2)
No hyperthermia	8 (44.4)	13 (61.9)	21 (53.8)
Prior surgery, n (%)			
Surgery	15 (83.3)	20 (95.2)	35 (89.7)
No surgery	3 (16.7)	1 (4.8)	4 (10.3)
Time on treatment (weeks)			
Median (range)	40.8 (25.2-58.2)	32.4 (22.4-40.9)	34.9 (23.8-48)
Cycles per patient, n (%)			
Median (range)	11 (8-14.8)	8 (6-12)	8 (6.5-14)
5 cylces	0 (0)	4 (19)	4 (10.3)
6 cylces	3 (16.7)	3 (14.3)	6 (15.4)
> 6 cycles	5 (27.8)	5 (23.8)	10 (25.6)
> 9 cycles	3 (16.7)	5 (23.8)	8 (20.5)
> 12 cycles	7 (38.9)	4 (19)	11 (28.2)
Total cycles			
N	207	214	421
Relative dose intensity, n (%)			
< 80%	43 (20.8)	31 (14.5)	74 (12.9)
≥ 80%	164 (79.2)	183 (85.5)	347 (87.1)

[a] Include two patients (11.1, Trabectedin+RHT) and one patient (4.8%, Trabectedin) with myxoid liposarcoma

[b] Multiple counts

Supplementary Table 1. Subgroup of patients ≥5 cycles and disease characteristics at baseline

Endpoint	Trabectedin+RHT *n* = 60	Trabectedin *n* = 58	Total *N* = 118	OR	95% CI	*p*-value
Full analysis set	*n* = 49	*n* = 46	*N* = 95			
**Best radiogical response**						
PR, *n* (%)	2 (4.1)	4 (8.7)	6 (6.3)	1.4	(0.66-2.99)	0.379
SD, *n* (%)	21 (42.9)	22 (47.8)	43 (45.3)			
PD, *n* (%)	26 (53.1)	20 (43.5)	46 (48.4)			
**Overall response**						
Yes, *n* (%)	2 (4.1)	4 (8.7)	6 (6.3)	2.17	(0.39-16.65)	0.39
No, *n* (%)	47 (95.9)	42 (91.3)	89 (93.7)			
**Disease control (PR + SD)**						
Yes, *n* (%)	23 (46.9)	26 (56.5)	49 (51.6)	1.38	(0.64-3.03)	0.411
No, *n* (%)	26 (53.1)	20 (43.5)	46 (48.4)			

Supplementary Table 2. Response assessment by RECIST v. 1.1 (ITT population)
Proportional odds model is used to estimate overall response (OR) comparing the effect of Trabectedin+RHT with Trabectedin on best radiological response; logistic model is used to estimate OR for overall response and disease control, after stratification for histological type (L-sarcoma/non-L-sarcoma), surgery (yes/no), metastasis (yes/no), and ECOG (0/>=1).
There were 23 patients with missing radiological response values, which were imputed by the mode of non-missing values for effect estimate and significance test.
